# An Update on the Use of Molecularly Imprinted Polymers in Beta-Blocker Drug Analysis as a Selective Separation Method in Biological and Environmental Analysis

**DOI:** 10.3390/molecules27092880

**Published:** 2022-04-30

**Authors:** Aliya Nur Hasanah, Ike Susanti, Mutakin Mutakin

**Affiliations:** 1 Pharmaceutical Analysis and Medicinal Chemistry Department, Faculty of Pharmacy, Universitas Padjadjaran, Jalan Raya Bandung Sumedang KM 21 Jatinangor, Bandung 45363, Indonesia; ike.susanti@gmail.com (I.S.); mutakin@unpad.ac.id (M.M.); 2 Drug Development Study Center, Faculty of Pharmacy, Universitas Padjadjaran, Jalan Raya Bandung Sumedang KM 21 Jatinangor, Bandung 45363, Indonesia

**Keywords:** molecularly imprinting, solid phase extraction, beta-blocker, separation

## Abstract

Beta-blockers are antihypertensive drugs and can be abused by athletes in some sport competitions; it is therefore necessary to monitor beta-blocker levels in biological samples. In addition, beta-blocker levels in environmental samples need to be monitored to determine whether there are contaminants from the activities of the pharmaceutical industry. Several extraction methods have been developed to separate beta-blocker drugs in a sample, one of which is molecularly imprinted polymer solid-phase extraction (MIP-SPE). MIPs have some advantages, including good selectivity, high affinity, ease of synthesis, and low cost. This review provides an overview of the polymerization methods for synthesizing MIPs of beta-blocker groups. The methods that are still widely used to synthesize MIPs for beta-blockers are the bulk polymerization method and the precipitation polymerization method. MIPs for beta-blockers still need further development, especially since many types of beta-blockers have not been used as templates in the MIP synthesis process and modification of the MIP sorbent is required, to obtain high throughput analysis.

## 1. Introduction

Beta-blockers are a type of cardiovascular drug that help to reduce morbidity and death in patients with heart failure [1]. They are commonly prescribed to patients with hypertension and are also indicated for angina, arrhythmias, after myocardial infarction and for hyperadrenergic states. The drugs in the beta-blocker group are acebutolol; labetalol; alprenolol; metipranolol; atenolol; metoprolol; betaxolol; nadolol; bisoprolol; oxprenolol; bunolol; pindolol; carteolol; propranolol; carvedilol; sotalol; celiprolol; timolol; and esmolol [2,3,4,5]. Beta-blockers have a very narrow therapeutic range (10–100 ng/mL) and are toxic at high concentrations. Overdose of beta-blocker can significantly reduce the heart rate to a dangerously low level, leading to life-threatening situations [6]. Athletes can abuse these beta-blockers because of their anti-tremor and anti-anxiety effects, and the Anti-Doping Administration and Management System (ADAMS) therefore collects biological samples for the purpose of doping control. Beta-blockers have been identified as Adverse Analytical Findings (AAFs): 0.4% of total samples contained beta-blockers, with 39% bisoprolol, 33% propranolol, 11% carvedilol, 11% metoprolol, and 6% sotalol [7]. Thus, the World Anti-Doping Agency established that beta-blockers were prohibited in competitions for archery, automobiles, billiards, darts, golf, shooting, skiing/snowboarding, and underwater sports. Beta-blockers have also been prohibited in and out of competition for archery and shooting [5].

As the treatment of cardiovascular illnesses grows, the use of drugs such as β-blockers poses a possible risk to the environment, as the rapid growth in drug manufacturing within the pharmaceutical industry is accompanied by ineffective wastewater treatment, leading to drugs not being eliminated in wastewater [8]. As a part of the pharmaceutical industry, the hospital wastewater was a contamination source of beta-blockers and their metabolites. Metoprolol is the most widely prescribed β-blocker [8]. In China, the consumption of metoprolol increased from 27,961 kg (2011) to 63,837 kg (2015), whereas prescriptions of sotalol, propranolol, and atenolol were less than 4000 kg/years [9]. Metoprolol is the most frequently identified in environmental samples, along with propranolol, which has bioaccumulation potential [10,11]. In Germany, metoprolol has been detected at around 160–2000 ng/L in wastewater [11,12]. The appearance of beta-blockers in the environment will cause a harmful effect as they can disrupt testosterone levels in male organisms [13,14]. Therefore, it is important to know the level of beta-blockers in the environment. All the beta-blockers have a solubility in water of about <62 mg/L to several thousand mg/L, and around 1–75% of parent drugs were excreted in urine [15,16]. In humans, after administration of beta-blockers, they are absorbed, distributed, partially metabolized, and eventually excreted in large amounts in the urine in non-metabolized forms such as atenolol (>85%) and nadolol (100%). Bisoprolol is metabolized in both urine and feces at a similar rate, about 50%. Betaxolol and propranolol are mostly metabolized at a rate of over 80% [17,18,19]. Metoprolol is excreted mainly in the human body, is removed by up to 85% during oxidative metabolism of the liver, and is converted to metabolites of O-desmethylmetoprolol (O-DMTP), α-hydroxymetoprolol (α-HMTP), and metoprololic acid (MTPA). MTPA is the major compound excreted by the kidneys at about 60–65% [6,20], but this may also apply to other metabolites to present in the urine, but at a much lower concentration [6].

Beta-blockers also were used during animal transportation to prevent unexpected death caused by their sedation effect. The residue of these drugs can accumulate in animal tissue and can be harmful and poisonous in humans [21]. The determination of beta-blockers in animal tissue for animal food use was conducted by Sai et al. (2012), with the results showing that metoprolol was found in one sample of pork at a concentration of 3.5 μg/kg [22].

Based on the abovementioned problems, an analytical method is needed to measure beta-blocker drugs in urine, serum, human plasma, animal food, and water samples. For drug monitoring, concentrations of beta-blockers in biological samples are low, and the concentration maximum (C_max_) of atenolol 3.4 + 1.0 h after administration of 100 mg is 537.1 + 112.7 ng/mL [23]. Whereas, the C_max_ of carvedilol 6 h after administration was 6.93 ng/mg (carvedilol 8 mg) and 77.94 ng/mL (carvedilol 128 mg) [24]. Table 1 provides lists of the C_max_ of various beta-blockers in plasma. Therefore, an appropriate extraction method is needed to separate beta blockers from a complex matrix. Several extraction methods have been used to separate beta-blocker drugs in various samples, such as liquid–liquid extraction (LLE) [23,25,26], protein precipitation [27,28], ionic liquid-phase microextraction [29], flow membrane microextraction [30], stir bar sorptive extraction [31], solid-phase extraction (SPE) [22,32,33], and air-assisted liquid–liquid microextraction (AALLME) [34]. Other methods can be seen in Table 2.

It can be seen from Table 2 that many extraction methods are used to separate beta-blocker compounds from the matrix. The development of extraction methods and analytical methods is still being carried out to increase sensitivity; the methods developed can be used to detect beta-blockers with low concentrations (LOD between 0.130 and 10 ng/mL, Table 2), which indicates the possibility of levels in the sample being at low concentrations. Moreover, the majority of beta-blockers are given in racemic mixes containing 50% of the (R)- and 50% of the (S)-enantiomer [49]. Therefore, a selective extraction method is needed to separate the analyte from the matrix. SPE effectively extracts and purifies chemicals from complex matrices [50]. To improve the selectivity and sensitivity of SPE, molecularly imprinted polymers (MIPs) are currently being developed and employed as sorbents. MIPs create artificial receptors in polymers, with selectivity and specificity against a specified analyte, making them ideal for use in extraction procedures [51]. The MIP sorbents were obtained by synthesis that involves the interaction of functional monomer with template molecule in solution to develop a covalent or non-covalent interaction complex. Functional monomer provides the reactive functional group that forms covalently or non-covalently with the target molecule [52].

Until now, there has been no review that discusses the use of MIPs for beta-blocker extraction. It is essential to know how much progress has been made in the development of MIPs for beta-blocker extraction so that further development can obtain MIPs with high beta-blocker extraction efficiency. This review provides a current and future perspective of MIPs for the extraction of beta-blocker drugs in various samples.

## 2. Methods of Synthesis of Molecularly Imprinted Polymers for Beta-Blocker Drugs

MIPs are sorbents produced through the interaction of template molecules with functional monomers. The functional monomer is copolymerized with cross-linkers to produce a solid polymer. After polymerization, the polymer is washed to remove the template molecules, resulting in binding sites capable of recognizing the molecule template in terms of its size, shape, and chemical functional group [53]. The materials used in the synthesis of MIPs are the template molecule, functional monomer, cross-linker, initiator, and porogenic solvent [51]. MIPs have many advantages, including good selectivity, high affinity, ease of synthesis, high-stress resistance, and low cost [54]. Several methods have been developed to produce MIPs, such as bulk polymerization [55], suspension polymerization [56], precipitation polymerization [57], emulsion polymerization [58], and surface imprinting polymerization [59]. The methods conducted so far for the production of MIPs for beta-blockers include bulk, precipitation, surface, and in situ polymerization. MIPs for beta-blockers have been sold in the market; this product is SupelMIP^TM^ Beta-Blocker cartridges. Morante-Zarcero and Sierra used this product to detect propranolol, metoprolol, pindolol, and atenolol in natural waters. However, there was a problem during the MIP-SPE process: the leakage of template molecule that interfered with the accuracy and precision of the target analyte [60]. However, this product is no longer available in the market [61]. Table 3 provides information about the methods for synthesis of molecularly imprinted polymers that have been developed for beta-blocker drugs based on articles found from 2011 to 2022.

### 2.1. Bulk Polymerization Method

The bulk polymerization method is widely used to produce MIPs. The MIP is typically prepared by solution polymerization, followed by mechanical grinding of the resulting bulk polymer to produce small particles, which are then sieved into the necessary size ranges, typically in the micrometer range [62,63]. This method has many advantages, such as its simplicity, straightforward imprinting condition optimization [63], and the use of a small amount of porogenic solvent [62].

The molecularly imprinting technique using bulk polymerization has been developed to selectively extract atenolol, carvedilol, pindolol, and sotalol in serum and urine samples [62,64,65,66,67,68]. Based on the study conducted by Gorbani et al., the MIP for the selective removal of atenolol in human urine samples was synthesized through the bulk polymerization method, with the MIP sorbent being synthesized using the non-covalent molecular imprinting approach. The materials used were acrylic acid as a functional monomer, dichloroethane as a porogenic solvent, ethylene glycol dimethacrylate (EGDMA) as a cross-linker, dibenzoyl peroxide as an initiator, and atenolol as the molecule template. Once synthesized, the degree of swelling of the polymeric MIP was determined, because the size and form of the imprinted sites varies when the MIP sorbent swells to a certain degree. The degree of swelling has a significant impact on its target compound identification capabilities, and the decrease in selectivity of the MIP sorbent could, therefore, be caused by a change in the shape of the imprinted sites [65]. The results show that the MIP has a higher swelling value at pH ≥ 7.5, because the number of negative charges of the carboxylate groups in the polymeric sorbents is more significant at pH 7.5 than at pH 3.5–5.0 due to deprotonation of the carboxyl groups provided in the acrylic acid. The polymeric chains oppose each other much more strongly as the number of carboxylate groups grows and more solvent molecules penetrate the polymeric chains, resulting in increased swelling. In organic solvent, the swelling value was lower when dichloroethane was used as a solvent. The binding capacity and imprinting factor (IF) of the MIP were 3.77 and 4.18 mg/g, respectively [65]. Another study using MIPs for the extraction of atenolol was conducted by Pratiwi et al. using methacrylic acid as a functional monomer and butanol as the porogenic solvent. This sorbent’s binding capacity and IF were 7.804 and 2.87 ± 0.2 mg/g [64]. Based on the studies mentioned above, the MIP produced by Pratiwi et al. has a higher binding capacity than the MIP produced by Gorbani et al., whereas the IF of the MIP produced by Gorbani et al. is higher than the MIP produced by Pratiwi et al. The IF value is the ratio of the adsorption capacity of the imprinting polymer and the non-imprinting polymer (NIP) [69]. Based on this, the MIP produced by Gorbani et al. looks promising for future development, because the higher IF shows a higher specific interaction between atenolol and the functional monomer [70] and also shows the efficiency of the MIP over its non-imprinting polymer (NIP) [71].

Generally, the synthesized MIPs will be placed in a SPE cartridge, which is used as an extraction holder to extract analytes in samples [60,72]. However, in the study conducted by da Silva et al., pipette tips were used as the extraction holder for the MIP, rather than a cartridge, which is the most common media used for the extraction holder [66]. The pipette-tip molecularly imprinted polymer solid-phase extraction (PT-MIP-SPE) is a modification of the SPE method using a polypropylene volumetric pipette tip (1000 μL) as an extraction holder, with the sorbent being inserted into the volumetric pipette tip to perform the extraction process [73]. Compared with SPE, PT-MIP-SPE needs a smaller amount of sorbent and a smaller volume of solvent; thus increasing the extraction efficiency [74,75]. da Silva et al. synthesized a PT-MIP sorbent to extract carvedilol using the bulk polymerization method, using methacrylic acid as a functional monomer and chloroform as a porogenic solvent [66]. The interaction between methacrylic acid and carvedilol is a hydrogen bond; accordingly, a nonpolar solvent with a lower dielectric constant (such as chloroform) was used as the porogenic solvent. The MIP obtained was characterized with a scanning electron microscope (SEM), and the sorbent was shown to have irregular particles with a nonuniform size. When the PT-MIP was used as a sorbent in human urine samples spiked with carvedilol enantiomers, the recovery reached almost 100% to NIP and C18, which is a common sorbent used for sample preparation. Thus, the results showed that the PP-MIP could be used as an adsorbent in PT-MIP-SPE to extract the carvedilol enantiomer from human urine.

Several challenges need to be considered in the MIPs synthesis process to produce MIP with good analytical performance, including determining the best molar ratio between functional monomers and molecular templates and selecting porogenic solvents [66]. Theoretically, choosing them can be undertaken by conducting studies related to the thermodynamic properties of the interactions between functional monomers/molecular templates or functional monomers/templates/porogenic solvents [66]. However, the bulk polymerization method also has some drawbacks, as it is time consuming because of the grinding process to obtain smaller particles, produces a low yield, and it not ideal for scale-up preparation. More crucially, it can only produce an irregularly shaped sorbent with a broad particle size distribution, which is challenging to employ in many practical applications [75]. The grinding process not only needs a lot of time, but can also damage the binding site cavity and reduce the recognition ability of the template molecule and the selectivity of the sorbent [62].

Based on articles found from 2011 to 2021, there are 6 articles out of 15 that use the bulk polymerization method to synthesize MIPs for beta-blockers, which are presented in Table 4. MIP technology using the bulk polymerization method has been developed for beta-blocker drugs such as atenolol, carvedilol, pindolol, and sotalol. The functional monomers used in the studies are methacrylic acid, acrylic acid, itaconic acid, 4-vinyl pyridine, and acrylamide, and the MIP sorbent was used to extract analytes in human urine and serum samples.

### 2.2. Precipitation Polymerization Method

The precipitation method is one of the most extensively utilized polymerization processes in the production of MIPs. This method has been conducted to overcome the drawbacks of the bulk method [75]. In general, the polymerization process is the same as the bulk polymerization method, except for the use of a higher amount of porogenic solvent, and there is no grinding process. The sorbent synthesized using this method has a uniform particle size, because the process of generating polymer chains increases until they become significant enough to become insoluble in the mixed reaction. After the polymerization process, the sorbent polymer can be easily obtained by filtration or centrifugation, and the process requires less time than bulk polymerization [62].

Hasanah et al. synthesized an MIP to extract atenolol (AT-MIP) using this polymerization method [72]. The monomer used in the MIP synthesis must interact with the template molecule to form a specific complex, and itaconic acid was chosen as the functional monomer because the binding affinity between atenolol and itaconic acid (−2.0 kcal/mol) was higher than the binding affinity between atenolol and methacrylic acid (−1.5 kcal/mol), based on an in silico study. AT-MIP was synthesized using different porogenic solvents: a mixture of methanol and acetonitrile for AT-MIP-1 and methanol for AT-MIP-2 (the volume of porogenic solvent used was 350 mL). The results showed that AT-MIP-1 had a higher polymer binding affinity than AT-MIP-2. AT-MIP-1 also had good selectivity, with an IF of about 11.02, and the IF increased (23.43) when it was applied to a sample spiked with mixed β-blockers. Moreover, the recovery of atenolol was up to 93.65 ± 1.29% using AT-MIP-1, and the porosity of AT-MIP-1 was also high, showing that the polymer has a binding site that can recognize the size of atenolol [72].

Many analytes of interest are now primarily found in water; separation/pre-concentration must, therefore, take place in an aqueous environment, such as organic compounds in wastewater or body fluids [76]. However, the presence of water molecules can significantly disrupt the molecular recognition of MIPs when extracting analytes from aqueous samples, since water can form non-selective interactions with the recognition sites of the MIPs, lowering the method’s selectivity. Coating MIPs with hydrophilic groups by adding hydrophilic monomers at the end of the polymerization process is one way to avoid this behavior. This hydrophilic layer establishes hydrogen bonds with water, reducing the interference of the analyte–polymer complex from this solvent [77,78,79] Based on this, MIPs coated with hydrophilic groups have been developed by Santos et al. for the extraction of oxprenolol from urine samples [80]. Glycerol dimethacrylate (GDMA) and hydroxymethyl methacrylate (HEMA) were used to coat the MIP. The maximum adsorption capacity of the coated MIP for oxprenolol was 82.6 mg/g, compared with 67.1 mg/g for the coated NIP, showing that the sizeable interaction of oxprenolol with MIP was due to molecular recognition, while a nonspecific interaction might lead to the interaction between oxprenolol and NIP.

**Figure 1:**
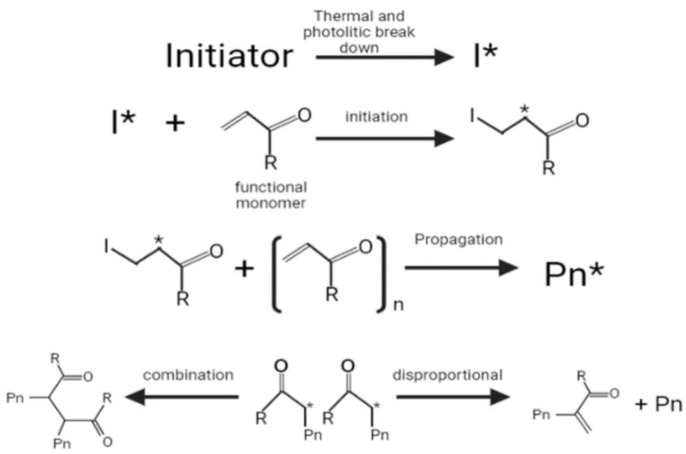
Radical polymerization: 1. Initiation; 2. Propagation; 3. Termination.

Radical polymerization is the most common method used to synthesize the MIPs, which requires the monomer and an initiator. The initiator is thermally or photolytically broken down into free radicals, and the π bond in the alkene monomer is then attacked by a radical, which forms a covalent bond with one of the carbon atoms while turning the other into a reactive radical (initiation stage) [81]. This is followed by a propagation stage, the latter continuously adding more monomers and growing into chains. The termination of chain growth ultimately occurs when a radical chain binds or participates in a disproportional reaction in which hydrogen is withdrawn from another radical chain (Figure 1) [81].

The combination of a functional monomer and an initiator has drawbacks, such as the tendency to generate non-imprinted polymer structures because the cross-linking monomer self-polymerizes and the formation of an imprinted molecular binding site when an active radical combines with a complex of a functional monomer and template [53]. To overcome these drawbacks, Liu et al. synthesized MIPs using a functionalized initiator for propranolol (PP-MIP) recognition, through the precipitation method [53]. The functional initiator is a radical chemical with the presence of functional end groups that can interact with the template molecule [82]; when the functionalized initiator was used, the functional monomer was not needed. The functionalized initiator was used to reduce the non-specific polymer obtained by the reaction between the cross-linker and the initiator, because the cross-linking reaction occurs around the bound molecule template, leading to the formation template. The functionalized initiator used in this study was 4,4′-azobis(4-cyanovaleric) acid (ACVA), because the carboxyl group of ACVA can form a hydrogen bond interaction with propranolol. The PP-MIP had an excellent maximum adsorption capacity (25.51 mg/g) compared to the non-imprinted polymer (PP-NIP). This study also synthesized MIPs using (S)-propranolol to produce a selective sorbent that can recognize the chiral molecule (sPP-MIP). The adsorption capacity of (S)-propranolol on the sPP-MIP (2.03 mg/g) was higher than that of (R)-propranolol (0.66 mg/g), showing that the binding site formed by (S)-propranolol was unable to accommodate (R)-propranolol due to the spatial distribution of its functional groups [53]. Table 5 presents several studies involving the synthesis of MIPs using the precipitation polymerization method.

To compare the effect of the bulk and precipitation polymerization methods, we compared the adsorption capacity, IF, and %recovery with the same template molecule (Table 6). Based on these studies, the adsorption capacity of the sorbent obtained using the bulk method was higher than that obtained using the precipitation method [62,64]. This could be because the total pore volume of the MIP from the precipitation technique may be reduced, and the particle porosity is high, which affects the extraction efficiency [84].

However, the IF of the sorbent obtained by the precipitation method was higher than that obtained by the bulk method [62,64]. The IF is the ratio of the adsorption capacity of the MIP to the adsorption capacity of the NIP [69]. An IF value of 1 implies that the MIP has no specific adsorption of the template molecule, because the template molecule recognition of the MIP is the same as that of the NIP [85], whereas an IF value of more than 1 shows that the template molecule recognition of the MIP is better than that of the NIP.

### 2.3. Surface Imprinted Polymerization

**Figure 2:**
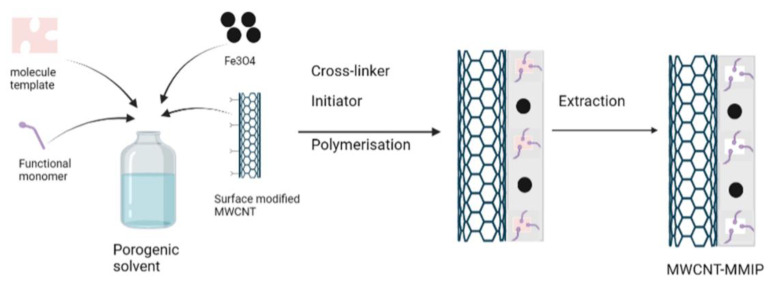
Scheme of synthesis of multiwalled carbon nanotubes based magnetic molecularly imprinted polymer (MWCNT-MMIPs).

MIPs using the bulk polymerization method have limitations, such as the uneven shape of the materials, low adsorption capacity, poor access to the binding site, sluggish mass transfer, inadequate template removal, and significant leakage of the template [86,87,88], and surface imprinted polymerization has been developed to overcome these limitation. In this method, the MIP shell anchors on the support material, such as fibers, graphene, silica particles, magnetite, multiwalled carbon nanotubes (MWCNTs), and inorganic substrates [89,90,91]. The surface imprinted method produces sorbents with uniform particle size, high specific surface, and better recognition, which increases the binding capacity and the fast mass transfer rate [92,93]. Ansari and Masoum synthesized a MWCNT-based magnetic molecularly imprinted polymer (MWCNT-MMIP) for the separation of sotalol in biological fluid [93]. MWCNTs have good properties, such as a large specific surface area, hollow structure, and high porosity, and the magnetic molecularly imprinted technique has been greatly developed. This method used an external magnetic field in the separation, because the sorbent has magnetic properties from the magnetic nanoparticles [94]. Magnetic nanoparticles are a type of nanoparticle (1–100 nm) with super-paramagnetism, unusual reactivity, and a huge specific surface area due to their nano size. Several nanoparticles, such as iron, nickel, cobalt, and their oxides, are employed as the core of the sorbents [95], with Fe_3_O_4_ being one of the most commonly used [96,97,98]. Magnetic separation has several benefits, such as its strength and flexibility, ease of the separation procedure because it eliminates the centrifugation and/or filtration process, high efficiency, and high adsorption [98]. The synthesis of the MWCNT-MMIP consisted of several steps: (1) synthesis of the magnetite nanoparticle, which was obtained through the solvothermal reduction method. FeCl_2_·4H_2_O and FeCl_3_·6H_2_O were dissolved in ultrapure water under stirring, the solution was heated, and the basic solution was added into the mixture solution under stirring; (2) pretreatment of the surface-modified MWCNTs to obtain carboxyl group-modified MWCNTs; (3) synthesis of MWCNT-MMIPs (Figure 2). Acrylamide was used as the functional monomer to produce the MWCNT-MMIP.

**Figure 3:**
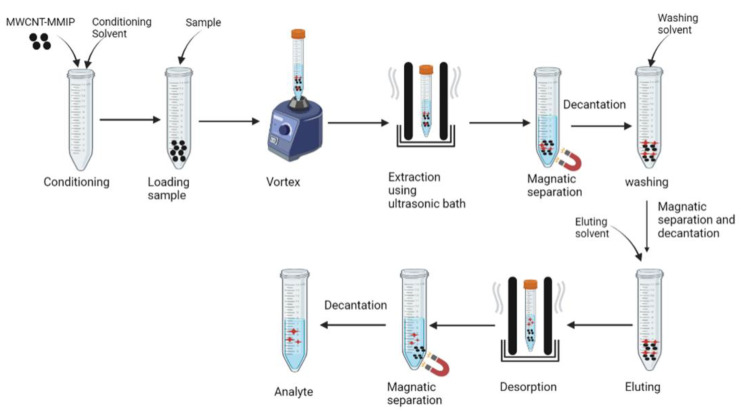
Procedure for extraction using ultrasonic-assisted dispersive solid-phase microextraction method (UA@DSPME).

The adsorption properties of the MWCNT-MMIPs have been evaluated. In the study adsorption isotherm, the regression coefficients of the Langmuir adsorption isotherm model, and Freundlich adsorption isotherm for MWCNT-MMIP are 0.9695 and 0.8898, respectively, showing that adsorption occurs at specific homogeneous sites in the adsorbent and forms a single layer (Langmuir isotherm) of sotalol on the surface of the absorbent. The maximum adsorption capacity of the MWNCT-MMIPs was 76.36 mg/g, which was higher than the non-imprinted polymer MWNCT-NMIPs (19.01 mg/g), showing that the MWNCT-MMIPs have a specific binding site that led to the excellent recognition of sotalol. The IF value of the MWNCT-NMIPs for sotalol has higher than for other compounds (IF sotalol 3.92, IF atenolol 1.29, IF propranolol 1.23, and IF nadolol 1.06), indicating that the MWNCT-NMIPs have high selectivity toward sotalol. The MWCNT-MMIPs were used as a sorbent in an ultrasonic-assisted dispersive solid-phase microextraction method (UA@DSPME) (Figure 3) to separate the sotalol in biological fluid. Human urine and plasma were spiked with sotalol standards, and the spiked human urine and plasma sample recoveries were 94.60–102.50% and 97.40–101.60%, respectively. These results show that the MWCNT-MMIPs can be applied as a good sorbent with excellent selectivity against sotalol because of their high surface area and specific imprinted site [93].

**Figure 4:**
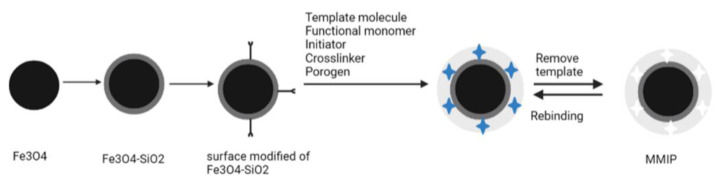
Schematic illustration of magnetic molecularly imprinted polymer (MMIP) synthesis.

Another study from Azodi-Deilami et al. made MMIPs for the selective separation of carvedilol (Figure 4) [99]. An Fe_3_O_4_ nanoparticle was used as the magnetic component, and this sorbent was used to extract carvedilol from human blood plasma samples. This approach detected carvedilol in the concentration range of 2–350 µg/L, with plasma sample detection and quantification limits of 0.13 and 0.45 µg/L, respectively. The recoveries reached between 85 and 93%.

### 2.4. In Situ Polymerization

**Figure 5:**
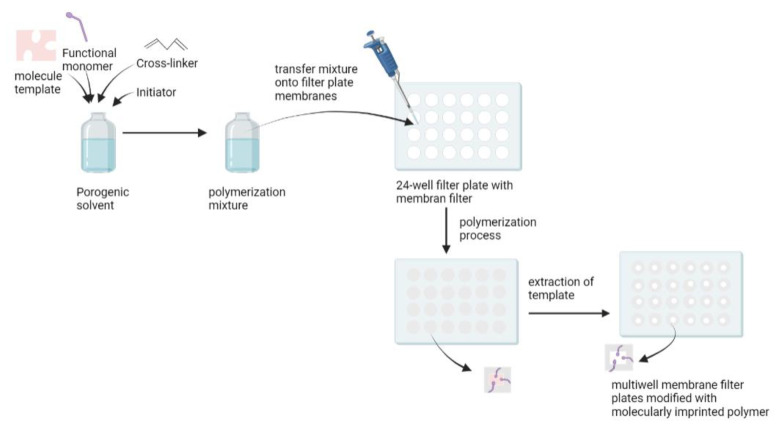
Multiwell membrane filter plates modified with MIPs.

An in situ polymerization method was developed to overcome the drawbacks of the conventional bulk method that requires crushing, grinding, and sieving to produce particulate MIPs, with the risk of destroying the specific binding cavity [100]. Renkecz et al. synthesized thin layer MIPs in multiwell membrane filter plates for the selective separation of propranolol [100]. Compared to standard SPE cartridges, the multiwell design allows for faster sample analysis and can be operated manually, in the same way as a syringe barrel using a vacuum manifold. It has the advantage of being able to handle samples in parallel, using multichannel pipettes. In this study, methacrylic acid was used as a functional monomer, oxprenolol as a dummy template, EDGMA as a cross-linker, and benzoin ethyl ether as the UV initiator. A mixture of these compounds was transferred to the filter plate membrane, and polymerization occurred under UV irradiation. The template was then removed from the sorbent (Figure 5). A dummy template was used because the levels of propranolol in the urine and serum were low, so leakage of the template could lead to false results. Therefore, the analogue structure approach was used [101]. The MIP-modified filter plates obtained were applied as a sorbent to separate propranolol in plasma and urine, with recovery values of 102% for urine samples and 97.2% for plasma samples [100].

A graphene oxide (GO)/MIP coated stir bar sorbent that is compatible with aqueous samples was prepared by Fan et al. through in situ polymerization [76]. The pre-polymer solution (a mixture of propranolol, GO, methacrylic acid, EGDMA, and Azobisisobutyronitrile (AIBN)) was prepared, then injected into a PTFE mold with a pretreated glass bar. Polymerization occurred at 60 °C for 24 h. After polymerization, an iron wire was inserted into the glass bar. To obtain a specific cavity, the propranolol template was removed by soaking in 8% acetic acid in methanol. The extraction procedure using the GO/MIP-coated stir bar was carried out by inserting the sample solution and GO/MIP-coated stir bar into a vial and then stirring. The GO/MIP-coated stir bar was then removed from the sample, washed, and dried. Subsequently, the GO/MIP coated stir bar was placed into a desorption solution and sonicated [76]. The extraction performance of the GO/MIP coated stir bar sorbent with a thickness of 700 µm was better than the GO/NIP-coated stir bar. Moreover, the GO in the MIP can increase the adsorption capacity of the GO/MIP coated stir bar, because GO has a high surface area and contains a functional group rich in oxygen. When this GO/MIP-coated stir bar sorbent was applied to the urine sample, the extraction efficiency reached 59.7% and the recovery values were 86.8–106% [76]. The method has some limitations, such as the time consuming synthesis and multistep preparation, and the adsorption being affected by the thickness of the MIP coating [76].

#### Monolithic Imprinted Polymerization

The monolithic imprinted polymerization method is a kind of in situ polymerization, which is a combination of the monolithic column and molecularly imprinted methods, used to increase selectivity [102]. Monolithic imprinted polymerization uses a simple, one-step in situ free-radical polymerization “molding” process directly within a chromatographic column. This method is not time consuming, because grinding, sieving, and column packing procedures are unnecessary [63]. Zhang et al. reported on a monolithic imprinted column for propranolol based on imprinting technology [103]. This column was used to concentrate propranolol from plasma samples. Hasanah et al. also carried out a study using a monolithic imprinted column for the separation of atenolol [102]. The components used in this study were methacrylic acid as functional monomer, atenolol as template molecule, propanol as a porogenic solvent, EGDMA as cross-linker, and benzoyl peroxide as radical initiator. The following steps make up monolithic imprinted polymerization:

1. The silanization process: a silicosteel column was hydrolyzed using acid and basic solution. Then, 3-(trimethoxysilyl)-propyl methacrylate was added to the hydrolyzed silicosteel column to start the salination reaction process. The reaction occurs between the methoxy and silanol groups of 3-(trimethoxysilyl)-propyl methacrylate on the wall surface of the silicosteel column. This reaction facilitated the formation of a covalent bond between the siliconsteel column and the monolithic polymer [104,105]
2. Synthesis of the imprinted monolithic column: the components used in the polymerization process were dissolved and inserted into the siliconsteel column by syringe, and both ends of the column were closed. The in situ polymerization occurred in an oven at 80 °C for 18 h [102].
3. Removal of template molecule using a mobile phase that can elute the atenolol. In the study conducted by Hasanah et al., a methanol:acetic acid (90:10 *v*/*v*) mobile phase at a flow rate of 0.01–0.03 mL/minute was used to remove the atenolol and residual compound [102].

An imprinted monolithic column with a template, monomer, and cross-linker ratio of 1:4:20 resulted in good inter and intracolumn reproducibility (RSD value 2.0%), and the imprinted monolithic column could be applied for the analysis of atenolol in biological samples (serum samples) with a recovery value of around 94.88 ± 4.43% [102].

As mentioned above, the MIP sorbents that developed were used for pre-treatment for beta-blockers analysis to have a good result. However, in its application, there are several challenges that need to be considered that might occur, such as the inhomogeneity of the active side of the sorbent which causes imprecise results, and leakage of template molecules so that several steps are needed to ensure that leakage does not occur, one of which is by using a method blank. To summarize the description of the various polymerization methods in MIPs synthesis for beta-blockers, Table 7 lists some of the advantages and disadvantages of each method.

## 3. Green Chemistry Principle in Molecularly Imprinted Polymer for Beta-Blockers

Green chemistry is a major motivation for laboratories and industries to promote sustainable growth. Green chemistry provides a workable basis to make chemical materials and processes more environmentally friendly (eco-friendly) [106]. The principles of green chemistry emphasize the need for safer, less toxic, and less harmful solvents or elimination of solvents, reduce energy consumption, avoid derivatization, and prioritize materials based on renewable resources [106,107].

Reducing the use of solvent in the extraction process also has a significant impact. The pipette-tip molecularly imprinted polymer solid-phase extraction (PT-MIPSPE) has already been developed to extract the carvedilol in the urine sample. In this technique, the solvent used was small; they used no more than 500 µL of solvent for each step in SPE [66]. On the other hand, the PT-MIP-SPE technique required a small amount of sorbent, use of 20 mg of sorbent was able to control the extraction process [66], and the mass amount is 10 times lower or more than the usual SPE technique.

The reusability and stability of MIP sorbent for beta-blockers have an important role in developing the green chemistry. The sorbent of MWCNT-based magnetic molecularly imprinted polymer (MWCNT-MMIP) for sotalol separation has been evaluated for reusability and the stability factor. The SPE process (adsorption–desorption) was repeated 12 times using the same sorbent. The result show that in the eight cycles, the adsorption capacity of sotalol declines slightly but is still above 90%, and it means the sorbent could be reused many times [93].

## 4. Conclusions

Several studies involving the synthesis of MIP have been discussed in this article. The synthesis of MIPs for the separation of beta-blocker drugs can occur using bulk polymerization, precipitation, monolithic imprinted polymerization, surface molecularly imprinted polymerization, and in situ polymerization. However, we cannot conclude which technique is the best because of the lack of studies conducted using different techniques but with the same ratio of composition. Based on the above studies, it can be seen that not all beta-blocker drugs have been used in the development of MIPs, as only drugs such as pindolol, atenolol, carvedilol, sotalol, propranolol, and oxprenolol have been used. Future studies involving MIPs for beta-blockers may need to focus on several areas, including:

1. Developing MIPs for separation or extraction using molecule templates that have not been used so far, such as acebutolol, labetalol, alprenolol, metipranolol, metoprolol, betaxolol, nadolol, bunolol, carteolol, celiprolol, timolol, and esmolol.
2. Studies about beta-blocker sensors, such as studies based on MIP technology are still lacking.
3. Developing an in situ polymerization technique, as MIPs modified in the multi-well membrane filter can be further developed to obtain high throughput analysis.
4. Developing MIPs for the extraction of beta-blockers in food samples, to obtain sorbents that are selective in their separation.

## Figures and Tables

**Table 1:** List of Cmax of various beta-blockers in plasma.

Name of Drug	Doses (mg)	C_max_ (ng/mL)	T_max_ (h)	Ref
Atenolol	100	537.1 + 112.7	3.4 + 1.0	[23]
Carvedilol	8	6.93	5.98	[24]
	128	77.94	6.02	
Bisoprolol	5	31	3	[35]
Metoprolol	80	100	1	[36]
Labetalol	200	182 ± 57 (fasting state); 180 ± 33 (after food)	1.42 ± 0.28 (fasting state); 2.08 ± 0.15 (after food)	[37]
Oxprenolol	80	22.5	1.21	[38]
Propranolol	40	24.9	2.1	[39]

Note: C_max_: measured peak plasma concentration; T_max_: time to reach peak concentration.

**Table 2:** Methods for the extraction of beta-blockers in different matrices.

Analyte	Sample	Extraction Method	Method	Linearities	LOD and LOQ	Ref
Atenolol	Plasma	LLE	HPLC with fluorescence detector	10–1000 ng/mL	NM	[25]
Atenolol	Plasma	LLE	HPLC with fluorescence detector	10–1000 ng/mL	NM	[23]
Atenolol	Urine	LLE	HPLC with fluorescence detector	5–150 ng/mL	1.5 ng/mL; 5.0 ng/mL	[26]
Propranolol	Plasma	Protein precipitation	HPLC with DAD detector	20–280 ng/mL	NM	[28]
Metoprolol	Serum	Protein precipitation	HPLC-MS/MS	5–250 ng/mL	NM	[27]
Bisoprolol				1–250 ng/mL		
Metoprolol	Urine and plasma	Continuous flow membrane microextraction	HPLC	5–700 µg/mL	1.0 ng/mL (LOD)	[30]
Propranolol				3–1000 µg/mL	0.5 ng/mL (LOD)	
Carvedilol	Urine, plasma, and tablet	Ionic liquid microextraction	Spectrofluorometer	0–250 μg/L	1.7 μg/L (LOD)	[29]
Celiprolol	Plasma	SPE	HPLC with fluorescence detector	1–1000 ng/L	NM	[32]
Carvedilol	Serum	Stir bar sorptive extraction	HPLC with UV detector	1.0–120.0 ng mL	0.3 and 1.0 ng/mL	[31]
23 compounds of β-Blockers	Animal food	SPE coupled with a clean-up step using methanol	HPLC coupled with linear ion trap mass spectrometry	5–200 μg/L	NM	[22]
Bisoprolol	Wastewater treatment plants	SPE	Liquid chromatography coupled with mass spectrometry (LC-MS/MS)	1–100 ng/mL	0.34–7.37 ng/L (LOQ)	[33]
Nadolol						
Betaxolol						
Atenolol						
Propranolol						
Pindolol						
Atenolol	Urine and plasma	AALLME using floating organic droplet solidification	UV-Vis spectrophotometry	0.30–6.00 μg/mL	0.30 μg/mL (LOQ)	[34]
Propranolol				0.30–1.40 μg/mL	0.26 μg/mL (LOQ)	
Carvedilol				0.30–2.00 μg/mL	0.30 μg/mL (LOQ)	
Timolol	Plasma	Cation-exchange SPE	Ion-pairing UPLC	5–300 ng/mL	1.7 ng/mL (LLOD); 5.0 ng/mL (LLOQ)	[40]
Metoprolol	Urine	A salting-out assisted liquid–liquid extraction (SALLE)	Hydrophilic interaction liquid chromatography-ultraviolet detection (HILIC-UV)	0.2–8.0 µg/mL	NM	[41]
Propranolol				0.1–4.0 µg/mL		
Carvedilol				0.1–4.0 µg/mL		
5-hydroxy carvedilol				0.2–8.0 µg/mL		
O-desmethyl carvedilol				0.1–4.0 µg/mL		
α-hydroxy metoprolol				0.2–8.0 µg/mL		
O-desmethyl metoprolol				0.2–8.0 µg/mL		
5-hydroxy propranolol				0.1–4.0 µg/mL		
Atenolol, metoprolol, esmolol, pindolol, and arotinolol	River water, influent wastewater (IWW), and effluent wastewater (EWW)	Magnetic solid phase extraction (MSPE)	Chiral LC-MS/MS	5–500 ng/mL	0.50–1.45 ng/L, 1.63–3.75 ng/L	[42]
21 β-blockers and 6 metabolites	Milk powder	Extracted using acetonitrile and purified with SPE	HPLC coupled with quadrupole orbitrap high-resolution mass spectrometry (HPLC-Q-Orbitrap HRMS)	0.5–500 µg/kg	0.2–1.5 µg/kg (LOD), 0.5–5.0 µg/kg (LOQ)	[43]
Atenolol	Human bone	SPE	Gas chromatography–mass spectrometry	0.1–150 ng/mg	0.1 ng/mL (LOD)	[44]
Bisoprolol				0–15 ng/mg	0.3 ng/mL (LOD)	
Atenolol	Rabbit plasma	SPE	Derivatization with hydrazonoyl chloride compound (UOSA54), determined using liquid chromatography–tandem mass spectrometry (LC-MS)	0.2–20.0 ng/mL	0.08 ng/mL, 0.20 ng/mL	[45]
Metoprolol						
Bisoprolol				0.2–18.0 ng/mL	0.05 ng/mL, 0.20 ng/mL	
Propranolol				0.1–15.0 ng/mL	0.03 ng/mL, 0.10 ng/mL	
Betaxolol				0.2–25.0 ng/mL	0.06 ng/mL, 0.25 ng/mL	
Metoprolol	Water	Vortex-assisted liquid–liquid microextraction based on in situ formation of a novel hydrophobic natural deep eutectic solvent (NADES-VA-LLME)	HPLC	1–100 μg/L	0.2 μg/L, 0.6 μg/L	[46]
Metoprolol	Plasma and urine	Magnetic dispersive micro-solid phase extraction	HPLC	5–10,000 ng/mL	0.8 ng/mL; 5 ng/mL	[47]
Atenolol				50–5000 ng/mL	10 ng/mL; 50 ng/mL	
Propranolol				10–5000 ng/mL	2 ng/mL; 10 ng/mL	
Atenolol	Plasma	LLME using a hydrophobic deep eutectic solvent	Gas chromatography-mass spectrometry (GC-MS)	0.064–5000 ng/mL	0.195 ng/mL, 0.645 ng/mL	[48]
Propranolol				0.043–5000 ng/mL	0.130 ng/mL, 0.435 ng/mL	
Metoprolol				0.069–5000 ng/mL	0.205 ng/mL, 0.692 ng/mL	

NM: not mentioned in the article; LOD: Limit of Detection; LOQ: Limit of Quantification; LLE: liquid–liquid extraction; HPLC: High-Performance Liquid Chromatography; DAD: Diode-Array Detection; HPLC-MS/MS: High-performance Liquid Chromatography–tandem Mass Spectrometry; AALLME: Air-Assisted Liquid–Liquid Microextraction; UPLC: Ultra-Performance Liquid Chromatography; SPE: Solid Phase Extraction.

**Table 3:** Synthesis methods of molecularly imprinted polymers that have been developed for beta-blocker drugs.

Beta-Blocker Drug	Synthesis Method
Atenolol	Bulk polymerization
	Precipitation polymerization
Carvedilol	Bulk polymerization
	Surface imprinted polymerization: magnetic molecularly imprinted polymer (MMIP)
Pindolol	Bulk polymerization
Sotalol	Bulk polymerization
	Surface imprinted polymerization: multiwalled carbon nanotubes based magnetic molecularly imprinted polymer (MWCNT-MMIP)
Propranolol	Precipitation polymerization
	In situ polymerization: thin layer MIPs in multiwell membrane filter plates
	In situ polymerization: graphene oxide (GO)/MIP coated stir bar sorbent
	Monolithic imprinted polymerization
Oxprenolol	Precipitation polymerization

**Table 4:** Studies involving the synthesis of MIPs using the bulk polymerization method.

Template	Monomer	Cross-Linker	Porogenic Solvent	Initiator	Q (mg/g)	IF	% Recovery	Application	Ref
Atenolol	Methacrylic acid	EGDMA	Butanol	Benzoyl peroxide	7.804	2.87 ± 0.2	NM	NM	[64]
Atenolol	Methacrylic acid	EGDMA	Propanol	Benzoyl peroxide	0.1043	2.872	66.54%	Extraction of atenolol in serum sample	[62]
			Butanol		7.804	2.868	32.22%		
Atenolol	Acrylic acid	EGDMA	Dichloro- ethane	Benzoyl peroxide	3.77	4.18	74.5–75.1%	Selective removal of atenolol in a human urine sample	[65]
Carvedilol	Methacrylic acid	EGDMA	Chloroform	4,4′-Azobis(4-cyanovaleric acid)	NM	NM	Around 100%	Used as adsorbent of PT-MIP-MS to extract carvedilol enantiomer in human urine	[66]
Pindolol	Itaconic acid	EGDMA	Acetonitrile	AIBN	125.76 *	2.27	NM	NM	[67]
	4-vinyl pyridine				9.93 *	1.89			
	Acrylonitrile				56.732 *	1.12			
Sotalol	Acrylamide	EGDMA	Dimethylformamide	AIBN	20.08	NM	97.4–102.5	Used as SPE sorbent for extraction of sotalol in urine sample	[68]

* The total number of binding sites μmol/g. NM: not mentioned in the article; Q: adsorption capacity; IF: Imprinting Factor; EGDMA: Ethylene Glycol Dimethacrylate; AIBN: Azobisisobutyronitrile; PT-MIP-SPE: Pipette-Tip Molecularly Imprinted Polymer-Solid Phase Extraction.

**Table 5:** Several studies on synthesis of MIPs using the precipitation polymerization method.

Template	Monomer	Cross-Linker	Initiator	Porogenic Solvent	Q (mg/g)	IF	% Recovery	Application	Ref
Atenolol	Itaconic acid	EGDMA	Benzoyl peroxide	Methanol: acetonitrile	4.250	11.02 (sample spiked with atenolol) 23.43 (sample spiked with mixed β-blocker)	93.65 ± 1.29%	Extraction of atenolol in serum sample	[72]
	Itaconic acid			Methanol	0.269	NM	NM		
Atenolol	Methacrylic acid	EGDMA	Benzoyl peroxide	Propanol	0.0804	11.721	74.64%	Extraction of atenolol in serum sample	[62]
				Butanol	2.950	4.160	10.86%		
Atenolol	Methacrylic acid	EGDMA	Benzoyl peroxide	Butanol	2.950	4.16 ± 2.1	NM	NM	[64]
Atenolol	Methyl methacrylate	EGDMA	Benzoyl peroxide	Butanol	2.166	5.967	NM	NM	[83]
Oxprenolol	Methacrylic acid	EGDMA	AIBN	Acetonitrile	82.6	NM	NM	Online MIP-SPE couple liquid chromatography and spectrometry conditions	[80]
(R,S) Propranolol	4,4′-Azobis(4-cyanovaleric) acid (functionalized initiator)	Trimethylolpropane trimethacrylate (TRIM)		Acetonitrile	25.51	NM	NM	Not mentioned in article, may be used to separate the chiral molecule in pharmaceutical product or others	[53]
(S)-Propranolol					2.03				

NM: not mentioned in the article; Q: adsorption capacity; IF: Imprinting Factor; EGDMA: Ethylene Glycol Dimethacrylate; AIBN: Azobisisobutyronitrile; MIP-SPE: Molecularly Imprinted Polymer-Solid Phase Extraction.

**Table 6:** Comparison of bulk and precipitation polymerization method in synthesis of MIP with atenolol as the template.

Template	Method	M	C	P	I	Q (mg/g)	IF	Ref
Atenolol	Bulk	Methacrylic acid	EGDMA	Butanol	Benzoyl peroxide	7.804	2.87 ± 0.2	[64]
	Precipitation					2.950	4.16 ± 2.1	
Atenolol	Bulk	Methacrylic acid	EGDMA	Butanol	Benzoyl peroxide	7.804	2.868	[62]
	Precipitation					2.950	4.160	
Atenolol	Bulk	Methacrylic acid	EGDMA	Propanol	Benzoyl peroxide	0.1043	2.872	
	Precipitation					0.0804	11.721	

M: functional monomer; C: cross-linker; P: porogenic solvent; I: initiator; m: homogeneity index of adsorption site; Q: adsorption capacity; IF: Imprinting Factor; EGDMA: Ethylene Glycol Dimethacrylate.

**Table 7:** Advantages and disadvantages of different polymerization methods.

Polymerization Methods	Advantages	Disadvantages
Bulk polymerization	Using a small amount of porogenic solvent. The size of MIP can be easily controlled.	Time-consuming because of the grinding process. Irregularly shaped sorbent Damage the binding site cavity and reduce the recognition ability because of the grinding process.
Precipitation polymerization	The regular shape of MIPs. Does not require grinding process. Easy procedure and less time consuming.	It needs a high amount of solvent. Precipitation occurs only when the polymer chains are large enough to be insoluble in the reaction mixture.
Surface imprinted polymerization	Uniform particle size. High specific surface. Better recognition increases the binding capacity. The fast mass transfer rate.	Needs a lot of steps in the synthesis.
In situ polymerization	Does not require crushing, grinding, and sieving to produce. The imprinted polymer is printed directly on the surface of the transducer or is immobilized after the synthesis process.	Removing template molecules often requires harsh conditions.

## Data Availability

Data sharing not applicable.
